# Associations between Ambient Particulate Matter and Nitrogen Dioxide and Chronic Obstructive Pulmonary Diseases in Adults and Effect Modification by Demographic and Lifestyle Factors

**DOI:** 10.3390/ijerph15020363

**Published:** 2018-02-19

**Authors:** Dirga Kumar Lamichhane, Jong Han Leem, Hwan Cheol Kim

**Affiliations:** 1 Department of Social and Preventive Medicine, College of Medicine, Inha University, Incheon 22212, South Korea; dirga_lamichhane@yahoo.com; 2 Department of Occupational and Environmental Medicine, College of Medicine, Inha University, Incheon 22212, South Korea; ekeeper21@naver.com

**Keywords:** adults, COPD, lung function, particulate matter, nitrogen dioxide

## Abstract

This study was undertaken to investigate the associations between chronic exposure to particulate matter of medium aerodynamic diameter ≤10 or ≤2.5 µm (PM_10_ or PM_2.5_) and nitrogen dioxide (NO_2_) levels and lung function and to examine a possible change in these relationships by demographic and lifestyle factors. Chronic obstructive pulmonary disease (COPD) was defined using the Global Initiative for COPD criteria (forced expiratory volume in 1 second (FEV1)/forced vital capacity (FVC) of <70%). Associations of lung function and COPD with PM_10_ or PM_2.5_ or NO_2_ were examined using linear and logistic regression analyses among 1264 Korean adults. The highest tertiles of PM_2.5_ (≥37.1 μg/m^3^) and NO_2_ (≥53.8 μg/m^3^) exposure were significantly associated with COPD (highest versus lowest tertile of PM_2.5_: adjusted odds ratio (OR) = 1.79, 95% CI: 1.02–3.13; highest versus lowest tertile of NO_2_: adjusted OR = 1.83, 95% CI: 1.04–3.21). A 10 μg/m^3^ increase in PM_10_ concentration was associated with a 1.85 L (95% CI –3.65 to –0.05) decrease in FEV1 and a 1.73 L (95% CI –3.35 to –0.12) decrease in FVC, with the strongest negative association among older people and those with less education. Reduced lung function was associated with PM_2.5_ exposure in subjects with no physical activity. This study provides evidence that exposure to ambient air pollution has adverse effects on lung function in adults.

## 1. Introduction

Chronic obstructive pulmonary disease (COPD) is a major cause of chronic morbidity and mortality and the fourth leading cause of death worldwide [1]. Cigarette smoking is generally considered the most important risk factor of COPD, although several other risk factors have been described [2]. Previous studies have associated air pollution, especially ambient particulate matter and nitrogen dioxide (NO_2_), with the prevalence of COPD and reduced lung function in adults [3,4,5]; a few studies have examined the effects of air pollution on objective measurements of lung function in adults [6,7]. The relationship between air pollution and lung function is further complicated by the existence of effect modifiers. Demographic and lifestyle factors have been reported to modify the association between air pollutants and lung function [8,9,10,11,12], but the findings remain inconsistent; some studies did not find any effect modification [4,13,14].

Some cross-sectional studies have reported links between traffic-related air pollution, such as particulate matter (aerodynamic diameter of ≤10 μm (PM_10_) and ≤2.5 μm (PM_2.5_)) and NO_2_, and COPD in adults, as defined by the Global Initiative for Chronic Obstructive Lung Disease (GOLD) criteria [15,16]. However, other cross-sectional studies reported no association between traffic-related air pollution and COPD prevalence [17,18]. Nevertheless, the majority of studies conducted on the relationship between traffic-related air pollution and lung function found that reduced lung function (a predictor of COPD mortality and morbidity) was associated with air pollution in adults [17,19], though others have failed to detect an association between air pollution and lung function [18,20]. Cohort studies have reported some evidence of an association between air pollution and COPD [21,22,23]. The European Study of Cohorts for Air Pollution Effects (ESCAPE) showed a significant positive association between air pollution and GOLD-defined COPD only in females [24]. Recent reviews of the literature have provided only suggestive evidence of associations between long-term exposure to air pollution and the prevalence and incidence of COPD (as defined by the GOLD criteria) among adults [2,23,25].

The limited number of studies and conflicting results highlight a need for more research on the relationship between air pollution and lung function in adults. Some studies conducted on this topic in Europe, North America, and China may not be applicable in Korea because of meaningful differences between health status characteristics, socioeconomic factors, and pollutant levels. In addition, it is also necessary and important to examine whether demographic and lifestyle factors modify the relationship between air pollution and lung function. Increased knowledge on the effect modification of these factors may lead to an enhanced understanding of an air pollution–health relation thought to have large population health impacts.

In this study, we investigated the long-term effects of ambient particulate matter (PM_10_ and PM_2.5_) and NO_2_ on lung function and COPD using the GOLD criteria. We further examined whether demographic and lifestyle factors (age, education, sex, smoking, physical activity, and body mass index) modify this relationship by a stratified analysis.

## 2. Materials and Methods

### 2.1. Study Population

This study was based on all available spirometry tests from the period January 2014 to December 2015 from a university hospital. When subjects visited the university hospital with respiratory symptoms and were suspected to have an underlying chronic respiratory condition (e.g., COPD or asthma), the subjects were suggested for spirometry testing. The study eligibility criteria were: an age between 20 and 85 years and residing in Seoul and Incheon (South Korea). Demographic information, lifestyle information, family history, and previous medical history were obtained using a self-administered questionnaire, and clinical data were obtained from medical records. A total of 1274 participants agreed to participate. We excluded participants with an active respiratory infection, an acute illness of any kind, or COPD (n = 9) or for whom no information on residential address was available (n = 1). Accordingly, the data of 1264 subjects were included in the analysis (Figure S1). Written informed consent was obtained from all participants after they had been fully informed of the survey protocol. The study protocol was approved by the Institutional Review Boards of Inha University Hospital.

### 2.2. Spirometry

Spirometry was conducted by a trained field technician using a portable microspirometer (Microspiro HI-298, Tokyo, Japan) according to the guidelines issued by the American Thoracic Society (ATS) [26]. COPD was defined using the GOLD criteria, that is, as a forced expiratory volume in 1 second (FEV1)/forced vital capacity (FVC) ratio of <70%.

### 2.3. Exposure Assessment

**Figure 1:**
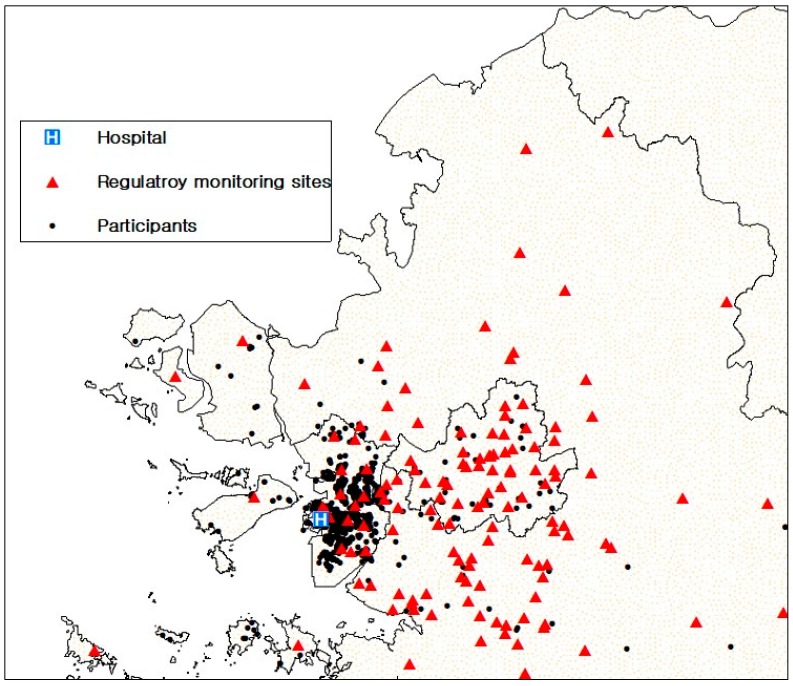
Study area and geocoding of population sample. Each dot on the map represents each subject’s home. The red-filled triangles indicate the distribution of regulatory monitoring stations.

Exposure to air pollution was assessed based on geo-coded residential addresses. Concentrations of PM_2.5_, PM_10_, and NO_2_ at residential addresses were estimated using land-use regression (LUR) models using a previously described standardized method [27]. In brief, we modeled air pollutants using LUR models and a regulatory monitoring network, and used ambient concentrations of PM_2.5_, PM_10_, and NO_2_ in the study area. Figure 1 shows the distribution of monitoring stations and study participants. We obtained hourly PM_10_, PM_2.5_, and NO_2_ concentrations measured at the 60 fixed monitoring stations in the study area (Seoul and Incheon) from the Korean Ministry of Environment (http://www.airkorea.or.kr/eng/information/stationInformation). There are four types of monitoring site in Korea: urban background, urban roadside, regional background, and national background. Measurements of air pollutants in our study were conducted at urban background monitoring sites.

Given the hourly measurements of PM_10_, PM_2.5_, and NO_2_, we computed daily averages for the days when the hourly measurements were recorded for more than 75% (18 hours) of the day. Then, the annual averages were calculated for the sites that had more than 75% (274 days) of daily data. We used centrally and locally available geographic variables as potential predictors. Predictor variables, such as traffic indicators, surrounding-land usage, topography, and spatial trends, were computed at each location using ArcGIS version 9.3 (a geographic information system) (ESRI, Redlands, CA, USA). Multiple linear regression models were built using a supervised forward stepwise procedure. Predictor variables used in the final LUR model for air pollution included the lengths of all roads within 300 m of residences, traffic intensity on nearest roads, total heavy-duty traffic loads of all roads within 100 m, urban green area within 300 m, and a variable representing spatial trends. The models explained 53–79% of the variability in measured PM_2.5_, PM_10_, and NO_2_ levels, depending on the validation method used, and the predicted values fitted well with the measured values, as reported in our previous study [28].

To assess the effects of exposure duration on COPD and lung function, we calculated one-year average concentrations for the year of the investigation (which was from 2014 to 2015) and three- and five-year average concentrations for the preceding three years and four years of the investigation, respectively.

### 2.4. Statistical Analysis

Initially, we performed a logistic regression analysis to examine associations between exposure variables (PM_2.5_, PM_10_, and NO_2_) and COPD. Then, we investigated relations between exposure variables and lung function outcome using linear regression; results are presented as β-values and 95% confidence intervals (CIs). Air pollution concentrations were entered as continuous variables without transformation and results are presented as change in outcome per 10 μg/m^3^ increase in exposure. For the logistic regression analysis, tertiles were calculated for each air pollutant level. Final models were adjusted for age (years), sex, education (<high school, high school, >high school), smoking status (current, former, non-smoker), body mass index (BMI), drinking status (yes and no), physical activity (yes and no), hypertension (yes and no), diabetes mellitus (yes and no), hyperlipidemia (yes and no), history of stroke (yes and no), family history of COPD (yes and no), family history of asthma (yes and no), and history of angina pectoris (yes and no). These covariates were selected based on previously reported results by applying a forward elimination procedure.

We also performed stratified analyses by age (<50th percentile and ≥50th percentile of age), BMI groups (50th percentile and ≥50th percentile), sex (male and female), regular physical activity (yes and no), smoking status (current, former, and non-smoker), and education (<high school and ≥high school). Effect modifications by these factors in association with each pollutant and lung function were calculated by including an interaction term. All analyses were performed using STATA 13.

## 3. Results

Table 1 shows the proportion of participants with each categorical potential risk variable and the adjusted odds ratios (ORs) and CIs for each variable. Of the 1264 study participants, 683 (54%) were female and 581 (46%) were male, and the average age was 57.9 years. We identified 94 participants (7.4%) with clinically diagnosed COPD based on a post-bronchodilator FEV1/FVC of <0.7. Older age and smoking appeared to be strongly associated with higher odds of COPD. People aged 70 years or older (adjusted OR (aOR) = 3.14 (95% CI: 1.25–7.91) showed an increased risk of COPD compared with people aged less than 50 years of age. Likewise, the risk of COPD was higher in current and former smokers compared with non-smokers. None of the other risk variables were statistically significantly associated with COPD.

The distributions of the one-, three-, and five-year mean particulate and NO_2_ air concentrations are summarized in Table 2. The ranges of pollutant concentrations represent data from all of the regulatory monitors combined. The mean concentration for PM_2.5_, PM_10_, and NO_2_ in the study areas during the study period appeared to be consistent. The five-year annual mean concentrations were 35.8 μg/m^3^, 53.6 μg/m^3^, and 45.6 μg/m^3^ for PM_2.5_, PM_10_, and NO_2_, respectively. The ranges of particulate matters were smaller than that of NO_2_. There were weak correlations between PM_2.5_ and PM_10_, but these particulate matters were not correlated with NO_2_.

Table 3 shows the ORs of the three-year mean particulate and NO_2_ air concentrations with COPD. The ORs for the annual mean and the five-year mean of these pollutants can be found in the supplementary file (Table S1). Logistic regression analysis showed that the highest tertile of NO_2_ exposure (three-year mean) was significantly associated with COPD, with aOR = 1.83 (95% CI: 1.04–3.21) for the highest tertile (≥53.8 μg/m^3^) compared with the lowest tertile (<41.0 μg/m^3^). Similar results were observed for PM_2.5_ (highest versus lowest tertile: aOR = 1.79 (95% CI: 1.02–3.13), whereas the result for PM_10_ was marginally significant (highest versus lowest tertile: aOR = 1.57 (95% CI: 0.92–2.69; *p* = 0.099). Furthermore, a 10 μg/m^3^ increase in NO_2_ concentration (three-year mean) appeared to be significantly associated with COPD (aOR = 1.14, 95% CI: 1.00–1.30 per 10 μg/m^3^ increase) and positive, but not statistically significant, associations were observed for PM_2.5_ and PM_10_. Results for the three-year mean of air pollutants did not change substantially when the pollutant levels were averaged for five-year periods. However, the dose-response relationship of the five-year mean of PM_2.5_ was not significant (*p* for trend =0.126). The association between PM_10_ and NO_2_ and COPD was stronger for the three- and five-year mean than for the annual mean, and the one-year average PM_2.5_ values showed variable associations with COPD (Table S1).

The results of linear regression analysis of the effects of PM_10_, PM_2.5_, and NO_2_ on lung function are shown in Table 4. In adjusted models, significant negative associations were observed between exposure to PM_10_ (3-year average) and FVC (β = –1.73; 95% CI: –3.35 to –0.12 per 10 μg/m^3^ increase in PM_10_) and FEV_1_ (β = –1.85; 95% CI: –3.65 to –0.05 per 10 μg/m^3^ increase in PM_10_). Likewise, an inverse, significant association was found between annual average PM_10_ level and FVC. NO_2_ and PM_2.5_ showed no significant associations with FVC or FEV_1_. Although COPD as defined by FEV1/FVC <0.7 was associated with higher levels of PM_2.5_ or NO_2_, none of the pollutants considered in this study demonstrated a significant association with the FEV_1_/FVC ratio.

We also examined the effects of air pollutant on lung function (FVC and FEV1) in different subgroups (Table 5). Age and education were potential modifiers of the association between PM_10_ exposure and FEV1 decline (*p* values for interactions <0.01). The adverse effect of PM_10_ on lung function was greater in participants aged ≥57 years than in those aged <57 years. Similarly, the effect of PM_10_ on lung function was greater among less-educated participants. To test the possibility of whether age and education were associated with FEV1 decline independent of exposure to PM_10_ in this study population, we constructed new models for education with and without interaction with age. In the adjusted model (adjusted for all confounders as described in the footnotes of Table 5) without an interaction term, lower level of education and older age were not associated with FEV1 decline. Furthermore, there was no interaction between education and age. This indicates that people with different levels of education and age did not differ in rate of FEV1 decline. Although interactions were not statistically significant, PM_10_ tended to have a stronger effect on FVC in male (β = –2.69; 95% CI: –4.92, –0.46) and former smokers (β = –3.61; 95% CI: –6.93, –0.29). Furthermore, the adverse effect of PM_2.5_ on lung function was greater among those that did not exercise.

**Figure 2:**
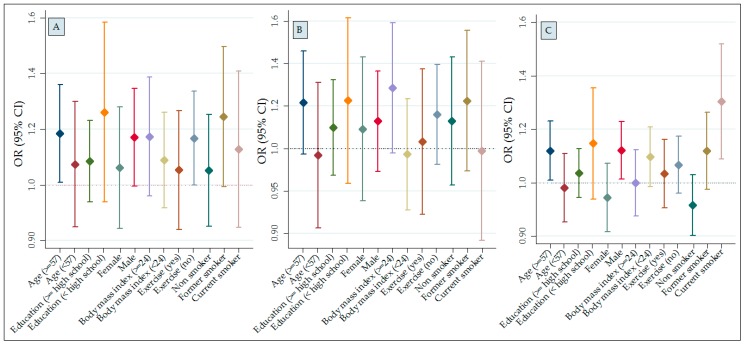
Associations of air pollution (3-year average) with COPD prevalence stratified by various characteristics. Solid diamonds indicate OR; vertical lines, 95% CI. Adjusted for confounders listed in Table 5 footnotes. Figure A presents associations for NO_2_ exposure, B presents associations for PM_10_ exposure, and C presents associations for PM_2.5_ exposure.

A plot of association of PM_10_, PM_2.5_, and NO_2_ with COPD prevalence in different subgroups appears in Figure 2. The association of COPD with NO_2_ and PM_2.5_ was greatest for older people. The PM_2.5_ model showed a stronger association with COPD for males. The association of PM_2.5_ with COPD was greatest in current smokers and was smaller in former and non-smokers. For PM_10_ and NO_2_, the associations showed the opposite pattern, but all 95% CIs were compatible with the null.

## 4. Discussion

In the present study, higher levels of PM_2.5_ and NO_2_ were significantly associated with increased COPD prevalence in adults using GOLD criteria. In addition, PM_10_ concentration showed significant negative associations with FVC and FEV_1_, and PM_10_ generally had stronger effects in older and less educated participants. The associations of COPD with PM_2.5_ were strongest in men, older adults, and in current smokers. FEV_1_ and FVC were not associated with PM_2.5_ and NO_2_ concentrations, and none of the three pollutants was associated with FEV_1_/FVC ratio.

Our findings that PM_2.5_ and NO_2_ were associated with the risk of GOLD-defined COPD in adults agree with those of other cross-sectional studies, which reported that COPD was positively associated with chronic exposure to PM and NO_2_ and living near a major road [3,15,16]. In an Italian study by Nuvolone et al. (2011), a positive association was reported between COPD and living within 100 m of a major road (aOR = 2.07, 95% CI: 1.11–3.87) in males only [16]. A study performed on 4757 women living in the Rhine-Ruhr Basin found that a 5-year mean increase of 16 μg/m^3^ in NO_2_ was significantly associated with the risk of COPD (OR = 1.39, 95% CI: 1.20–1.63), and that women living less than 100 m from a busy road were 1.79 times (95% CI: 1.06–3.02) more likely to have COPD than those living farther away [15]. A recent study in China reported that elevated PM_2.5_ levels were probably associated with the prevalence COPD (adjusted OR 2.42, 95% CI: 1.42–4.12, for >30 and ≤75 μg/m^3^ compared with a lower level of ≤35 μg/m^3^) [3]. Furthermore, our finding of a nonsignificant positive relationship between PM_10_ and COPD concurs with that of a previous study, in which exposure to PM_10_ non-significantly increased the risk of COPD [15]. On the other hand, some cross-sectional studies found no association between air pollution and COPD in adults [17,18].

Available evidence strongly supports that chronic air pollution reduces lung function in children, but the majority of such studies in adults have been conducted on susceptible populations [19,29]. Our finding that PM_10_ has an adverse effect on lung function in adults is in line with the observations that FVC and FEV_1_ are negatively associated with traffic-related air pollution [3,5,9,24,25]. In a recent ESCAPE study, an association was observed between exposure to PM_10_ and NO_2_ and reduced FVC and FEV [19], but average levels of PM_10_ (25 μg/m^3^) were much lower than those observed in the present study (51–54 μg/m^3^). Furthermore, as was reported in two previous studies [17,18], we did not observe a significant association between NO_2_ level and any spirometric parameter. In addition, no association was observed between any of the three pollutants and a reduction in FEV1/FVC ratio, as has been previously reported [12,15,30,31]. Some of the discrepancies between reported associations between air pollution and COPD or lung function may have been due to different exposure assessment durations [32].

The observed interactions between PM_10_ and age and education on lung function (FEV_1_ and FVC) agree with observations made in recent studies, which reported that age and education modify the relationship between air pollution and respiratory health [33,34]. Studies have also demonstrated an association between age and lung function, and suggested that an advanced age is associated with reduced lung function [11]. The effect of air pollution (PM_10_ or ozone) on lung function is more prominent among the elderly [35,36], and thus PM_10_ and age act synergistically to reduce lung function. This may be due to a cohort effect or older people being more susceptible to air pollution effects on lung function. Our finding that education modified relationships between PM_10_ or PM_2.5_ and lung function is supported by Cakmak et al. (2016), who found that a lower education increased lung function impairment resulting from exposure to PM_10_ [33]. Moreover, among our subjects that did not exercise, PM_2.5_ appeared to have stronger negative effects on lung function, which suggests that physical activity has a beneficial effect. This finding is consistent with that of a previous study, which investigated the protective effect of physical activity on particulate air pollution-induced pulmonary response [8].

As would be expected, current smokers had the strongest association between COPD and PM_2.5_. The finding of the strongest association between FVC and PM_10_ among former smokers is unable to be described. Although it was unlikely to be due to subjects with lung disease giving up smoking, it is possible that former smokers with COPD may retain a propensity to the adverse effect of PM_10_.

The mechanism responsible for the effect of chronic exposure to air pollution on COPD in adults is unknown, but oxidative stress and pulmonary inflammation have been suggested to be main mechanisms in animal studies [37,38]. We found that NO_2_ and PM_2.5_ were associated with COPD prevalence. NO_2_ is considered to be an airway irritant that potentially interacts with the immune system to cause respiratory tract infections and promote lung inflammation [39]. Chronic exposure to PM_2.5_ may have an irritative effect on airways and trigger inflammatory response in lung tissue [40]. It has also been suggested that reactive oxygen species and free radicals generated in response to NO_2_ and PM_2.5_ can overwhelm the redox system and damage cell-wall lipids, proteins, and DNA, thereby inducing airway inflammation [41].

The present study has three strengths: 1) the exposure variables used were computed using geographic information system (GIS) tools at participants’ addresses, whereas postcodes have been used in many previous studies; 2) An objective definition of COPD was used; and 3) We evaluated the potential effect modifier by a stratified analysis, and all results were adjusted for many potential confounders. However, the study also has its limitations. First, the ideal definition of COPD remains controversial [42,43]. In this study, we used the GOLD definition (FEV1/FVC <0.7), which is considered as a key guideline in the management of COPD. However, the accuracy of lung function criteria for the diagnosis of COPD in the elderly has been questioned [44]. This may lead to an overdiagnosis of COPD in very old people. Nevertheless, previous studies have suggested that COPD may be either overdiagnosed or underdiagnosed depending on the approach taken to defining abnormal lung function [43,45]. In addition, in our study, the diagnosis of COPD is based on a single initial spirometry test, as stated in the current international clinical COPD guideline [46]. A recent study found that a substantial proportion of subjects (15.1%) shifted diagnostic category (obstructed/non-obstructed) by 1 year when using spirometry (fixed ratio) as a cut-off [47]. Future studies need to consider the short-term or long-term consistency of a spirometric diagnosis of airway obstruction while examining the association of air pollution with COPD in this study population. Second, our exposures were estimated by an LUR model. Although LUR models provide fine-scale spatial estimates for air pollution [48], they are subject to the spatial effects associated with the properties of spatial data. LUR models may give extremely high or low predictions when the distribution of selected variables across residences falls outside the range of the monitoring sites [49]. However, we used 60 sampling locations to develop the LUR models. The inclusion of these locations would have increased exposure contrast. Hoek et al. [48] suggested a minimum of 40–80 sampling locations in order to properly specify an urban LUR model. Third, our exposure variables were based on current home addresses, and thus some degree of exposure misclassification may have occurred when subjects moved to other locations. However, this probably only affected a limited number of individuals and resulting misclassifications were likely to be nondifferential. Fourth, despite the extent of the adjustment performed for multiple covariates, such as demographic characteristics, lifestyle, health-related factors, and cigarette smoking, unmeasured factors, such as levels of aeroallergens (pollen, fungus, and dust mites), may have confounded the effect of air pollutants on lung function. However, as our study is restricted to non-asthmatic adults, the potential confounding effect of airway sensitization is minimal. Finally, the cross-sectional nature of the present study precludes definitive statements on causal relationships.

## 5. Conclusions

Chronic exposure to PM_2.5_ and NO_2_ was found to be associated with higher COPD prevalence in adults. The results obtained also suggest that exposure to particulate air pollution is associated with reduced lung function, that PM_10_ exposure is associated with lower FVC and FEV_1_ values, and that the effects of PM_10_ exposure are greater in the elderly and in those with a low level of education. Furthermore, an association was observed between PM_2.5_ exposure and lower FVC and FEV_1_ in subjects that did not exercise. On the other hand, NO_2_ was not found to influence lung function in all participants or by a stratified analysis. Further research is needed to determine whether pollutant types differentially affect lung function.

## Figures and Tables

**Table 1:** Potential risk variables for study participants (n = 1264).

Variable	n (%) or mean ± SD	COPD (n)	Prevalence (%)	Adjusted * OR (95% CI)
Sex				
Female	683 (54.03)	30	4.39	Reference
Male	581 (45.97)	64	11.02	1.20 (0.53–2.74)
Age, years				
<50	225 (17.80)	12	5.33	Reference
50–59	527 (41.69)	31	5.88	1.36 (0.66–2.78)
60–69	380 (30.06)	37	9.74	2.69 (1.25–5.78)
≥70	132 (10.44)	14	10.61	3.14 (1.25–7.91)
Education				
<High school	341 (26.98)	22	6.45	Reference
High school	503 (39.79)	43	8.55	1.39 (0.77–2.50)
>High school	420 (33.23)	29	6.90	1.07 (0.56–2.02)
BMI				
<18.5	17 (1.34)	1	5.88	Reference
18.5–25	799 (63.21)	69	8.64	0.81 (0.10–6.51)
>25	448 (35.44)	24	5.36	0.42 (0.05–3.53)
Smoking				
Non-smoker	796 (62.97)	34	4.27	Reference
Former smoker	291 (23.02)	34	11.68	2.52 (1.09–5.81)
Current smoker	177 (14.00)	26	14.69	4.33 (1.84–10.22)
Drinking				
No	672 (53.16)	40	5.95	Reference
Yes	592 (46.84)	54	9.12	1.01 (0.59–1.72)
Exercise				
Yes	528 (41.77)	33	6.25	Reference
No	736 (58.23)	61	8.29	1.19 (0.74–1.91)
Hypertension				
No	872 (68.99)	64	7.34	Reference
Yes	392 (31.01)	30	7.65	0.77 (0.46–1.29)
Diabetes mellitus				
No	1119 (88.53)	78	6.97	Reference
Yes	145 (11.47)	16	11.03	1.42 (0.76–2.65)
Hyperlipidemia				
No	954 (75.47)	66	6.92	Reference
Yes	310 (24.53)	28	9.03	1.37 (0.81–2.30)
History of stroke				
No	1239 (98.02)	92	7.43	Reference
Yes	25 (1.98)	2	8.00	0.87 (0.19–4.02)
Family history of COPD				
No	1255 (99.29)	92	7.33	Reference
Yes	9 (0.71)	2	22.22	3.97 (0.70–22.51)
Family history of asthma				
No	1237 (97.86)	94	7.60	
Yes	27 (2.14)	0	0	NS
History of angina pectoris				
No	1211 (95.81)	89	7.35	Reference
Yes	53 (4.19)	5	9.43	0.80 (0.29–2.20)
FVC, L (meanness)	86.69 ± 12.69			
FEV1, L (mean ± SD)	90.40 ± 14.17			
% FEV1/FVC	80.63 ± 8.27			
COPD (FEV1/FVC)				
<0.7	94 (7.44)			
≥0.7	1170 (92.56)			

* Adjusted for age, sex, education, smoking status, body mass index (BMI), drinking status, physical activity, hypertension, diabetes mellitus, hyperlipidemia, history of stroke and angina pectoris, and family history of chronic obstructive pulmonary disease (COPD) and asthma. NS, non-significant; OR, odds ratio; FVC, forced vital capacity; FEV1, forced expiratory volume in 1 second.

**Table 2:** Distributions of PM_2.5_ PM_10_ and NO_2_.

Length of exposure	Pollutant (μg/m^3^)	Mean (SD)	Median	IQR	Range
5 years	PM_2.5_	35.82 (5.49)	37.07	6.64	14.94–46.5
	PM_10_	53.56 (4.40)	52.81	5.53	39.18–69.21
	NO_2_	45.63 (17.68)	49.83	18.38	3.34–118.94
3 years	PM_2.5_	33.83 (5.62)	35.22	7.05	14.49–46.24
	PM_10_	50.98 (4.30)	50.17	5.44	38.87–67.32
	NO_2_	44.64 (17.65)	48.6	18.87	3.34–135.87
1 year	PM_2.5_	33.39 (6.05)	34.97	7.26	14.52–48.08
	PM_10_	50.68 (4.57)	49.53	6.39	39.56–73.34
	NO_2_	44.81 (17.75)	49.17	18.05	3.50–132.97

PM_2.5_, particulate matter with an aerodynamic diameter of ≤2.5 μm; PM_10_, particulate matter with an aerodynamic diameter ≤10 μm; IQR, interquartile range.

**Table 3:** Logistic regression analyses of crude and adjusted associations between air pollutants (3-year averages) and COPD (n = 1264).

Air Pollutants *	COPD	
	Crude	Adjusted ^†^
	OR (95% CI)	OR (95% CI)
NO_2_ (μg/m^3^)		
Lowest tertile (<41.0)	Reference	Reference
Medium tertile (≥41.0 and <53.8)	1.28 (0.73–2.25)	1.39 (0.77–2.52)
Highest tertile (≥53.8)	1.93 (1.14–3.27)	1.83 (1.04–3.21)
*p* for trend		0.012
Per 10 μg/m^3^ increase	1.13 (0.99–1.27)	1.14 (1.00–1.30)
PM_10_ (μg/m^3^)		
Lowest tertile (<48.5)	Reference	Reference
Medium tertile (≥48.5 and <52.2)	1.17 (0.68–2.01)	1.23 (0.70–2.15)
Highest tertile (≥52.2)	1.51 (0.90–2.54)	1.57 (0.92–2.69)
*p* for trend		0.113
Per 10 μg/m^3^ increase	1.38 (0.87–2.21)	1.39 (0.85–2.25)
PM_2.5_ (μg/m^3^)		
Lowest tertile (<32.7)	Reference	Reference
Medium tertile (≥32.7 and <37.1)	1.43 (0.82–2.48)	1.54 (0.86–2.75)
Highest tertile (≥37.1)	1.77 (1.04–3.02)	1.79 (1.02–3.13)
*p* for trend		0.035
Per 10 μg/m^3^ increase	1.32 (0.88–1.97)	1.34 (0.89–2.02)

* Air pollutant concentrations are three-year averages. ^†^ Adjusted for age, sex, education, smoking status, body mass index, drinking status, physical activity, hypertension, diabetes mellitus, hyperlipidemia, history of stroke and angina pectoris, and family history of COPD and asthma.

**Table 4:** Associations between air pollutants (10 μg/m^3^) and lung function (n = 1264).

Parameter	Length of exposure	Air Pollutant (μg/m^3^)	Crude	Adjusted *
			β (95% CI)	β (95% CI)
FVC (l)	5 years	NO_2_	0.35 (−0.05, 0.75)	0.18 (−0.22, 0.59)
		PM_10_	−1.38 (2.97, 0.21)	−1.52 (−3.10, 0.05)
		PM_2.5_	−0.61 (−1.89, 0.66)	−0.62 (−1.89, 0.65)
	3 years	NO_2_	0.33 (−0.06, 0.73)	0.18 (−0.22, 0.58)
		PM_10_	−1.52 (−3.14, 0.11)	−1.73 (−3.35, −0.12)
		PM_2.5_	−0.83 (−2.08, 0.41)	−0.85 (−2.10, 0.39)
	1 year	NO_2_	0.27 (−0.13, 0.66)	0.11 (−0.28, 0.52)
		PM_10_	−1.46 (−2.99, 0.07)	−1.77 (−3.29, −0.25)
		PM_2.5_	−0.80 (−1.96, 0.36)	−0.85 (−2.01, 0.30)
FEV_1_ (l)	5 years	NO_2_	0.19 (−0.25, 0.63)	0.11 (−0.33, 0.56)
		PM_10_	−1.58 (−3.36, 0.19)	−1.73 (−3.50, 0.03)
		PM_2.5_	−0.81 (−2.24, 0.61)	−0.86 (−2.28, 0.56)
	3 years	NO_2_	0.19 (−0.25, 0.64)	0.13 (−0.32, 0.58)
		PM_10_	−1.62 (−3.44, 0.19)	−1.85 (−3.65, −0.05)
		PM_2.5_	−0.83 (−2.22, 0.57)	−0.94 (−2.33, 0.45)
	1 year	NO_2_	0.15 (−0.29, 0.59)	0.09 (−0.36, 0.53)
		PM_10_	−1.13 (−2.84, 0.58)	−1.46 (−3.16, 0.24)
		PM_2.5_	−0.78 (−2.07, 0.51)	−0.96 (−2.25, 0.32)
FEV_1_/FVC	5 years	NO_2_	−0.07 (−0.32, 0.19)	−0.05 (−0.29, 0.19)
		PM_10_	0.05 (−0.99, 1.08)	0.09 (−0.93, 1.10)
		PM_2.5_	0.00 (−0.83, 0.84)	−0.09 (−1.03, 0.85)
	3 years	NO_2_	−0.04 (−0.30, 0.22)	−0.03 (−0.28, 0.21)
		PM_10_	0.11 (−0.95, 1.17)	0.16 (−0.89, 1.21)
		PM_2.5_	0.08 (−0.73, 0.90)	0.02 (−0.89, 0.93)
	1 year	NO_2_	−0.04 (−0.30, 0.22)	−0.03 (−0.27, 0.21)
		PM_10_	0.28 (−0.72, 1.28)	0.40 (−0.57, 1.37)
		PM_2.5_	0.00 (−0.75, 0.76)	−0.00 (−0.85, 0.85)

* Adjusted for age, sex, education, smoking status, body mass index, drinking status, physical activity, hypertension, diabetes mellitus, and a history of stroke, COPD, and asthma.

**Table 5:** Changes in FVC and FEV_1_ associated with a 10 μg/m^3^ increase in pollutants (3-year average) (n = 1264).

Parameter	Stratified Characteristics	Air Pollutants		
		NO_2_	PM_10_	PM_2.5_
		β (95% CI)	β (95% CI)	β (95% CI)
FVC	Age			
	<57	−0.31 (−0.87, 0.24)	−0.97 (−3.19, 1.25)	−1.63 (3.33, 0.01)
	≥57	0.48 (−0.05, 1.01)	−2.39 (−4.67, −0.11)	−0.57 (2.41, 1.28)
	*p* for interaction	0.545	0.089	0.252
	Sex			
	Male	0.21 (−0.36, 0.79)	−2.69 (−4.92, −0.46)	−0.85 (−2.72, 1.02)
	Female	0.14 (−0.36, 0.63)	−0.98 (−3.27, 1.32)	−1.04 (−2.75, 0.68)
	*p* for interaction	0.530	0.403	0.787
	Body mass index			
	<24	−0.03 (−0.64, 0.58)	−2.25 (−4.64, 0.15)	−0.66 (−2.52, 1.20)
	≥24	0.29 (−0.23, 0.82)	−1.33 (−3.48, 0.83)	−1.35 (−2.99, 0.29)
	*p* for interaction	0.943	0.993	0.434
	Exercise			
	Yes	0.19 (−0.39, 0.76)	−1.68 (−4.35, 0.99)	−0.11 (−2.01, 1.79)
	No	0.19 (−0.34, 0.72)	−1.75 (−3.83, 0.34)	−1.76 (−3.49, −0.04)
	*p* for interaction	0.653	0.796	0.171
	Education			
	<High school	0.59 (−0.19, 0.14)	−4.26 (−7.27, −1.26)	−0.87 (−3.73, 1.99)
	≥High school	0.03 (−0.42, 0.49)	−0.77 (−2.62, 1.09)	−0.82 (−2.22, 0.58)
	*p* for interaction	0.193	0.065	0.939
	Smoking			
	Current smoker	0.31 (−0.76, 1.39)	−2.53 (−6.95, 1.92)	−0.15 (−3.23, 2.93)
	Former smoker	0.00 (−0.82, 0.82)	−3.61 (−6.93, −0.29)	−0.31 (−3.27, 2.65)
	Non-smoker	0.27 (−0.23, 0.77)	−1.43 (−3.45, 0.58)	−1.57 (−3.16, 0.02)
	*p* for interaction	0.935	0.815	0.412
	Age			
	<57	−0.27 (−0.80, 0.26)	0.18 (−1.99, 2.35)	−0.21 (−1.97, 1.54)
	≥57	0.33 (−0.25, 0.90)	−3.42 (−6.12, −0.72)	−1.89 (−4.03, 0.25)
	*p* for interaction	0.870	0.007	0.273
	Sex			
	Male	0.18 (−0.50, 0.86)	−2.64 (−5.20, −0.01)	−0.64 (−2.69, 1.41)
	Female	0.05 (−0.51, 0.61)	−1.07 (−3.68, 1.53)	−1.41 (−3.34, 0.53)
	*P* for interaction	0.564	0.482	0.581
	Body mass index			
	<24	−0.08 (−0.73, 0.58)	−1.34 (−3.94, 1.26)	−1.28 (−3.29, 0.72)
	≥24	0.23 (−0.38, 0.84)	−2.49 (−4.99, 0.01)	−1.11 (−3.02, 0.80)
	*p* for interaction	0.902	0.276	0.853
	Exercise			
	Yes	0.04 (−0.57, 0.65)	−1.95 (−4.87, 0.98)	0.24 (−1.79, 0.23)
	No	0.19 (−0.40, 0.79)	−1.57 (−3.89, 0.76)	−2.03 (−3.95, −0.10)
	*p* for interaction	0.976	0.971	0.107
	Education			
	<High school	0.63 (−0.33, 1.59)	−5.99 (−9.66, −2.32)	−3.03 (−5.98, −0.09)
	≥High school	−0.03 (−0.54, 0.47)	−0.23 (−2.29, 0.18)	−0.03 (−1.59, 0.15)
	*p* for interaction	0.201	0.008	0.092
	Smoking			
	Current smoker	−0.01 (−1.25, 1.14)	−1.87 (−6.78, 3.04)	−2.51 (−5.89, 0.87)
	Former smoker	0.16 (−0.65, 0.97)	−3.51 (−7.48, 0.46)	−0.45 (−3.54, 2.65)
	Non-smoker	0.21 (−3.00, 0.72)	−1.60 (−3.88, 0.68)	−1.02 (−2.81, 0.78)
	*p* for interaction	0.709	0.839	0.660

All models were adjusted for age, sex, education, smoking status, body mass index, drinking status, physical activity, hypertension, diabetes mellitus, and a history of stroke, COPD, asthma, and angina pectoris. Stratified characteristics were not included as confounders.

## Supplementary Materials

The following are available online at http://www.mdpi.com/1660-4601/15/2/363/s1, Figure S1. Flow chart of the study population, Table S1: Logistic regression analyses for crude and adjusted associations of air pollution with COPD.
