# Supplementary material for: Associations between Ambient Particulate Matter and Nitrogen Dioxide and Chronic Obstructive Pulmonary Diseases in Adults and Effect Modification by Demographic and Lifestyle Factors
Source: Int J Environ Res Public Health. 2018 Feb 19;15(2):363. doi: 10.3390/ijerph15020363 (PMC5858432; doi:10.3390/ijerph15020363)
Supplement: Supplementary file 1 [file ijerph-15-00363-s001.docx]

**Supplementary Material: Associations between Ambient Particulate Matter and Nitrogen Dioxide and Chronic Obstructive Pulmonary Diseases in Adults and Effect Modification by Demographic and Lifestyle Factors**

Dirga Kumar Lamichhane, Jong Han Leem and Hwan Cheol Kim *


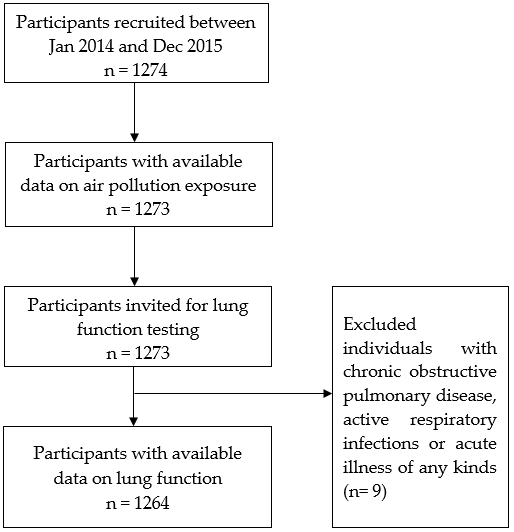


**Figure S1.** Flow chart of the study population.

**Table S1.** Logistic regression analyses for crude and adjusted associations of air pollution with COPD.

| **Length of Exposure** | **Air Pollutant** | **Crude** | **Adjusted *** |
| --- | --- | --- | --- |
|  |  | **OR (95% CI)** | **OR (95% CI)** |
| 5 years | NO_2_ (μg/m^3^) |  |  |
|  | Lowest tertile (<42.6) | Reference | Reference |
|  | Medium tertile (≥42.6 and <54.6) | 1.29 (0.73–2.30) | 1.32 (0.72–2.42) |
|  | Highest tertile (≥54.6) | 2.13 (1.25–3.62) | 2.01 (1.14–3.55) |
|  | *P* for trend |  | 0.042 |
|  | Per 10 μg/m^3^ increase | 1.13 (0.99–1.28) | 1.14 (1.00–1.30) |
|  | PM_10_ (μg/m^3^) |  |  |
|  | Lowest tertile (<51.2) | Reference | Reference |
|  | Medium tertile (≥51.2 and <54.7) | 1.07 (0.62–1.86) | 1.10 (0.62–1.95) |
|  | Highest tertile (≥54.7) | 1.62 (0.97–2.70) | 1.61 (0.95–2.75) |
|  | *P* for trend |  | 0.159 |
|  | Per 10 μg/m^3^ increase | 1.32 (0.83–2.09) | 1.34 (0.83–2.16) |
|  | PM_2.5_ (μg/m^3^) |  |  |
|  | Lowest tertile (<34.8) | Reference | Reference |
|  | Medium tertile (≥34.8 and <38.9) | 1.27 (0.73–2.22) | 1.38 (0.77–2.46) |
|  | Highest tertile (≥38.9) | 1.74 (1.03–2.94) | 1.77 (1.02–3.06) |
|  | *P* for trend |  | 0.126 |
|  | Per 10 μg/m^3^ increase | 1.36 (0.75–2.48) | 1.41 (0.93–2.14) |
| 1 year |  |  |  |
|  | NO_2_ (μg/m^3^) |  |  |
|  | Lowest tertile (<41.5) | Reference | Reference |
|  | Medium tertile (≥41.5 and <53.9) | 1.32 (0.76–2.29) | 1.38 (0.78–2.46) |
|  | Highest tertile (≥53.9) | 1.70 (1.00–2.88) | 1.62 (0.92–2.83) |
|  | *P* for trend |  | 0.242 |
|  | Per 10 μg/m^3^ increase | 1.12 (0.99–1.27) | 1.14 (1.00–1.30) |
|  | PM_10_ (μg/m^3^) |  |  |
|  | Lowest tertile (<48) | Reference | Reference |
|  | Medium tertile (≥48 and <52.1) | 0.83 (0.48–1.44) | 0.83 (0.47–1.47) |
|  | Highest tertile (≥52.1) | 1.34 (0.81–2.20) | 1.34 (0.80–2.25) |
|  | *P* for trend |  | 0.210 |
|  | Per 10 μg/m^3^ increase | 1.18 (0.75–1.84) | 1.15 (0.72–1.83) |
|  | PM_2.5_ (μg/m^3^) |  |  |
|  | Lowest tertile (<32.5) | Reference | Reference |
|  | Medium tertile (≥32.5 and <36.8) | 1.67 (0.98–2.87) | 1.84 (1.05–3.24) |
|  | Highest tertile (≥36.8) | 1.52 (0.88–2.63) | 1.51 (0.85–2.67) |
|  | *P* for trend |  | 0.104 |
|  | Per 10 μg/m^3^ increase | 1.20 (0.83–1.72) | 1.21 (0.83–1.75) |

* Adjusted for age, sex, education, smoking status, body mass index, drinking status, physical activity, hypertension, diabetes mellitus, hyperlipidemia, stoke, family history of COPD, and angina pectoris.

© 2018 by the authors. Submitted for possible open access publication under the
terms and conditions of the Creative Commons Attribution (CC BY) license (http://creativecommons.org/licenses/by/4.0/).
